# Impact of agricultural interventions on the nutritional status in South Asia: A review

**DOI:** 10.1016/j.foodpol.2016.05.002

**Published:** 2016-07

**Authors:** Vijay Laxmi Pandey, S. Mahendra Dev, Usha Jayachandran

**Affiliations:** Indira Gandhi Institute of Development Research, Gen. Vaidya Marg, Goregaon (East), Mumbai, India

**Keywords:** Agricultural intervention, Nutrition, South Asia, Women empowerment

## Abstract

Nearly half of the malnourished population of the world lives in South Asia, and agriculture is the main source of livelihood of the people in this region. Many review exercises have analysed the available evidences to understand the ways in which agriculture can be leveraged to enhance nutritional status; however, very few of them have employed a systematic approach ensuring internal and external validity. The present paper seeks to fill this gap for strengthening the policy recommendations. It demonstrates an association between agricultural interventions and nutritional outcomes; and it shows that the production of targeted nutrition-rich crops, homestead gardens, and diversification of the agricultural production system towards fruits and vegetables and aquaculture can potentially improve nutrient intake and nutritional outcomes. The empowerment of women and nutrition knowledge play a crucial role in establishing linkage between agriculture and nutritional outcomes.

## Highlights

• Home production of targeted crops for nutrients intake and nutritional outcomes.
• Homestead gardens for improved diet diversity.
• Diversification of agriculture for improved diet diversity and nutritional outcomes.
• No conclusive evidence of impact of irrigation on diet diversity.
• Empowerment of women crucial for improving nutritional status.

## 1 Introduction

The South Asian (SA) region is home to nearly half of the poor and malnourished population of the world. In this region, food insecurity is high, with almost 23% of the population not having access to adequate calorie intake (WDI, 2014). Agriculture is the main source of livelihood as it employs 60% of the total workforce (FAO, 2013). The potential of agriculture for producing nutritious food is not appropriately tapped for reducing the malnourishment in this region. Production of quality food in adequate quantity alone may not improve nutritional outcomes, unless malnutrition is addressed by adopting a multi-sectoral approach (FAO, 2013, Das et al., 2014). Therefore, the agricultural policies and programmes need to be more nutrition-sensitive for improving the nutritional outcomes (FAO, 2013).

There are multiple pathways through which agriculture can influence the nutritional outcomes (UNICEF, 1990, Hawkes and Ruel, 2006, Van Dorp et al., 2011, Gillespie et al., 2012, Webb, 2013). The UNICEF framework, illustrating pathways, was built on the understanding ‘food alone is not enough’ and it has conceptualized linkages between agriculture and nutrition. Subsequently these linkages have been modified for specific pathways and to generate testable hypotheses (Headey et al., 2012, Kadiyala et al., 2014), as the following:

1. *Source of food:* Agricultural produce by the farmers is also used for their own household food consumption.
2. *Source of income of households engaged in agriculture, for food and non-food expenses:* Agricultural income may be earned either through wages earned as agricultural workers or through the sale of produce. The income spent on nutritious food may have impact on malnutrition.
3. *Agricultural policy and food prices:* Agricultural policies can affect the relative prices and affordability of various marketed food and non-food crops.
4. *Women in agriculture and their socio-economic status:* The socio-economic status of women in intra-household decision making and resource allocation may influence the nutritional status of the mothers and their children.
5. *Maternal employment in agriculture, child care and feeding:* The involvement of mothers in agriculture may influence their ability to manage child care and feeding.
6. *Women in agriculture, maternal nutrition, and health status:* The maternal nutritional outcomes and health may be compromised due to involvement in agriculture. The work-related energy expenditure may exceed the intake, or the dietary diversity may be compromised. Some of the agricultural practices may be hazardous to their health. These factors, consequently, affect the nutritional status of the children.

These pathways are considerably influenced by different factors such as the type of agriculture, market and consumer demands, inequalities in the system, tastes and preferences, and nutrition-relevant policies and programmes. Therefore, the pathways from agriculture to nutrition are evolving and dynamic; and are not linearly associated (Kadiyala et al., 2014).

In the recent past, several studies have focused on leveraging or modifying agriculture to enhance nutrition. Many review exercises have analysed the available evidences on the linkages between agriculture and nutritional outcomes. Some of them are global in nature and others have country specific focus. In the SA region, women constitute approximately 35% of the agricultural workforce. In the rural areas of this region, 70% of the employed women are engaged in agricultural sector, indicating the importance of agriculture (FAO, 2011). However, women face more severe constraints in accessing production resources, markets, and services than those faced by men. Because of high dependence on agriculture for livelihood and the role of agriculture for women in this region, all the aforementioned pathways are crucial for promoting improved nutritional outcomes through nutrition-sensitive agricultural interventions.

The studies on agricultural interventions, some of them based in the SA countries, have focussed on several factors including increasing production of micronutrient-rich food (Berti et al., 2004, Burchi et al., 2011, Gibson, 2011, Masset et al., 2011, Potts and Nagujja, 2007, Webb et al., 2012), diet diversification (Arimond et al., 2011, Haider and Bhutta, 2008, World Bank, 2007), animal based food (Kawarazuka, 2010), homestead gardens (Girard et al., 2012, Haddad and Meeker, 2013, Ruel, 2001). Webb and Kennedy (2014) have combined evidence from 10 review papers and shown the linkages between agriculture and nutrition on a global level. Kadiyala et al. (2014) and Yosef et al. (2015) have mapped the available evidences on linkages between agriculture and nutrition, in India and Bangladesh, respectively.

The studies focusing on agricultural interventions to improve production of micronutrient-rich foods, through increased productivity, home gardening, bio-fortification, diversification, etc., have reported a significant impact on dietary diversity and micronutrient intake, through the first and/or second pathways. Policies which affect the relative price of staples (third pathway) may also have considerable effect on the nutritional intake. However, no conclusive evidence exists on the impact of these interventions on nutritional outcomes (Berti et al., 2004, Haddad and Meeker, 2013, Masset et al., 2011, Ruel, 2001, World Bank, 2007). It has been pointed out that the insignificant impact could be attributed to poor evidence and a lack of methodological rigour in the study design, evaluation and analysis (Arimond et al., 2011, Berti et al., 2004, Kadiyala et al., 2014, Leroy and Frongillo, 2007, Masset et al., 2011, Ruel, 2001, Webb and Kennedy, 2014). The evidences on the fourth, fifth, and sixth pathways are even more limited (Haddad and Meeker, 2013, Kadiyala et al., 2014, Yosef et al., 2015).

The interventions directly affecting multiple pathways, through nutrition-education and multi-sectoral aspects of the involvement of women, considerably improved the nutritional outcomes (Berti et al., 2004, Webb and Kennedy, 2014). Therefore, a holistic approach involving more than a single sector and focusing on education, knowledge enhancements including nutrition education, nutrition counselling, and empowerment of women can immensely contribute in tackling the micronutrient deficiencies (Girard et al., 2012, Haider and Bhutta, 2008, Ruel, 2001).

The existing review papers show that agriculture does have the potential to promote nutritional outcomes, although the linkages are rather complex (Haddad and Meeker, 2013, Kadiyala et al., 2014). The reviews have emphasized the need for understanding the pathways and synergies between agriculture and nutrition on the basis of various contextual drivers such as the governance of food systems, climate change, competing demands for key resources, and change in value chain (Berti et al., 2004, Haddad and Meeker, 2013, Webb and Kennedy, 2014, World Bank, 2007). The need for extensive research on appropriate metrics and relevant methodologies has also been emphasized (Berti et al., 2004, Haen et al., 2011, Kadiyala et al., 2014, Masset et al., 2011, Ruel, 2001, Webb and Kennedy, 2014).

Several reviews have reported the inter-linkages between agricultural interventions and nutritional outcomes; however, few have used a systematic approach (Berti et al., 2004, Haider and Bhutta, 2008, Kadiyala et al., 2014, Kawarazuka, 2010, Leroy and Frongillo, 2007, Masset et al., 2011). The selection of empirical papers on the basis of internal and external validity has not been very common. Furthermore, most of these reviews have been limited to a particular programme or country (Kadiyala et al., 2014, Kawarazuka, 2010, Leroy and Frongillo, 2007). The aim of the present paper is to conduct a systematic review to assess the evidence base in the SA region. It analyses existing evidences for combating food insecurity and malnutrition through agricultural interventions. The studies for review are selected on the basis of internal and external validities through the assessment of the quality considering the study design and methodology used. The next section of the paper presents the methodology for selection of the studies. The review results are presented in the third section, followed by discussion in the fourth section and conclusions in the last section.

## 2 Methodology

We analysed the studies on the impact of interventions in agriculture and allied sectors (horticulture, livestock, fisheries and forestry) on the nutritional outcomes for adults and children, published since the year 2000. The nutritional outcomes were captured through both intermediate outcome indicators such as dietary diversity, calorie intake and nutrient intake, and outcome indicators such as anthropometric factors and DALYs (Disability Adjusted Life Years). The process for selection of the published studies and the criteria adopted for assessment of the internal and external validities is given in the following two subsections.

### 2.1 Study search and screening

The search was conducted for shortlisting the empirical studies with agricultural interventions or programmes as the central concern. A systematic literature search was carried out using several search engines and websites of relevant institutions: Google Scholar, AgEcon Search, CAB Abstracts, EconLit, Eldis-IDS, Food and Agriculture Organization (FAO), International Food Policy Research Institute (IFPRI), Government of India (Planning Commission, Ministry of Agriculture, Ministry of Women and Child Welfare), Leveraging Agriculture for Nutrition in South Asia (LANSA), Ingenta Connect, Harvest Plus, United Nations Children’s Fund (UNICEF), and World Bank. We have not considered grey literature and the literature available in languages other than English. A wide range and combination of catch phrases, with terms ‘impact/effect’, ‘agriculture/agricultural’, ‘interventions/programmes’, ‘nutrition’, ‘nutritional outcomes’, and ‘South Asia’ were used, on the literature published during the period of January 2000 to July 2014. The term ‘health’ was not included in the search process as it resulted in an unmanageably large number of irrelevant results. To reduce the prospect of missing relevant publications on agricultural programme and policies due to limitations of the used search phrases, the search was repeated in many related publication databases. Table 1 shows the number of papers obtained using different search phrases in Google Scholar.

The search results from Google Scholar were scanned by using the advanced search with all words ‘agriculture nutrition South Asia’ and the exact phrase ‘agriculture nutrition’ until the relevance of the search title diminished. The search yielded in 2080 papers (Table 1). On the basis of the title and relevance of the first 1000 results, 129 papers were identified for comparison with results from other search engines and relevant websites to check for duplication and omission. Nineteen more papers were added to this identified set, including few global studies and a study from Vietnam (Ogle et al., 2001). On the basis of the abstracts, 32 of these 148 papers were found to be unrelated to our theme and hence were excluded. The remaining116 papers were examined for the study design and analytical approach. It was observed that 30 of them were review exercises and 61 were either conceptual papers or presented the facts without analysis, or did not relate agricultural interventions or programmes with nutrition. After excluding these 91 papers, our review narrowed down to a set of 25 studies which used empirical analysis.

### 2.2 Systematic review

For conducting a systematic review, a set of twenty-five studies were selected as described in the previous section. The set was not homogenous in terms of a common outcome indicator and the studies used different metrics for examining the linkage. Some studies analysed nutritional outcomes by examining the stunting and wasting of children and adolescent, whereas others analysed the Body Mass Index (BMI) for determining adult malnutrition or levels of micronutrients such as vitamin A and haemoglobin. Some studies used intermediate outcome indicators, such as changes in consumption patterns, dietary diversity, and intakes of certain foods. Such differences in techniques and indicators made comparisons across studies difficult and limited the scope of the aggregation of results into a single metric, as also indicated by Masset et al. (2011) and Webb and Kennedy (2014). The selected studies were then classified as low, medium, or high quality on the basis of internal validity and also on the basis of external validity. The model validity was assessed by scoring the empirical analysis for internal validity on the basis of counterfactual analysis. For external validity, the studies were assessed on the basis of the programme theory and heterogeneity of impact as shown in Table 2.

**Fig. 1:**
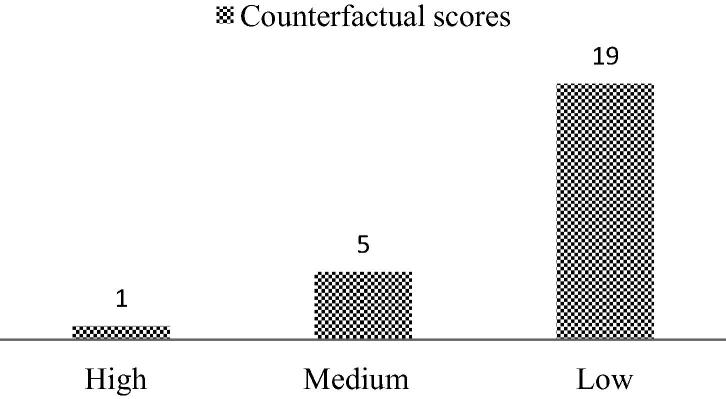
Internal validity scores.

Most of the included empirical studies analysed data from secondary datasets and had large sample sizes. Others were primary baseline surveys, with smaller sample sizes. None of the included studies used randomized control trials (RCT). The power test for sample size selection was not reported in these studies; and hence they could not be scored on the basis of their power calculations in our analysis. With the studies showing large variations in sample sizes and many not adhering to sampling procedures, a direct comparison of the results was not feasible. Based on our scoring criteria for counterfactual methods, only one study (Ogle et al., 2001) was scored as high, five studies were scored as medium (Ghosh, 2007, Hanji, 2006, Jahan and Pemsl, 2011, Kiresur et al., 2010, Weinberger, 2005), and the remaining 19 studies were scored as low (Fig. 1 and Table 3). As the similarities between the counterfactuals were extremely low, ex-post power calculation to assess the ability of the studies to bring forth nutritional impacts of agricultural interventions was not possible.

**Fig. 2:**
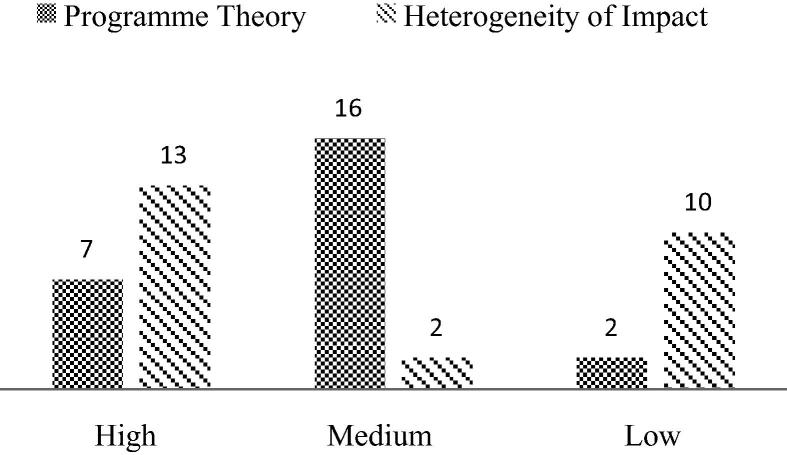
External validity scores.

External validity (Pelletier, 2002) is established to reveal the conceptual framework and causal relationships which can then be extrapolated to other areas with different geographic and socio-economic characteristics. The heterogeneity of the effects based on population characteristics, including socio-economic attributes of the population and intermediate outcomes is important for understanding the underlying causal relationships. In terms of scoring for external validity, only the studies pertaining to nutritional outcomes, namely, anthropometric outcomes and DALYs, were scored for both programme theory and intermediate outcome analysis. Papers pertaining to changes in consumption patterns and women empowerment were scored for the programme theory alone because these indicators themselves are intermediate development outcomes in the pathways that connect agriculture with nutrition. These studies were also scored for the heterogeneity of impact. For programme theory, seven studies were scored as high, 16 were scored as medium and two were scored as low. On the parameter of the heterogeneity of impacts, the scores were high for 13 studies, medium for two, and low for 10 (Fig. 2 and Table 3).

The reviewed empirical studies scored better on external validity than on internal validity. A large number of studies presented a sound programme theory in terms of conceptual frameworks. Some studies also analysed intermediate outcomes, making their external validity sound. In terms of heterogeneity of impact, many studies analysed causal effects of varying population characteristics, such as socio-economic conditions and regional variations, lending more credibility to their findings. Thus, the studies on linkages between agricultural interventions and nutritional outcomes have duly emphasised the underlying programme theory, analysing intermediate outcomes and heterogeneity of impact (Table 3).

## 3 Results

Afghanistan, Bangladesh, Bhutan, India, Maldives, Nepal, Pakistan, and Sri Lanka constitute the SA region. Of the 25 studies selected in this review, 10 were based on India, six on Bangladesh, three on Nepal, and one on Pakistan. No study based on Afghanistan, Bhutan, Maldives, and Sri Lanka was shortlisted for the review. Moreover, we included one study based on Vietnam and four global studies covering South Asian countries. Therefore, there exists a regional imbalance in the coverage of the SA countries. Evidences in these studies for the different pathways are examined in the following subsections to understand how effectively agricultural interventions can be used to improve the nutritional outcomes.

### 3.1 Pathway 1: Sources of food

Twenty-two of the 25 reviewed studies examined the contribution of agriculture as a source of food for nutrition. The studies indicate strong evidence that the dietary intake of agricultural households largely depends on food supplies from their own farm (Parasuraman and Rajaretnam, 2011, Yu, 2012), because subsistence farming is common in this region. The evidence is not conclusive for the impact of own supply on livestock based food consumption (Roos et al., 2003, Bhagowalia et al., 2012).

Agricultural productivity can be improved by providing timely access to inputs, improvement of rural and marketing infrastructure (Gaiha et al., 2012), and adherence to timelines. A negative and significant association has been reported between improvements in agricultural productivity and under-nutrition (Gulati et al., 2012, Shively et al., 2012, Webb and Block, 2012). Particularly, the interventions for increasing the productivity and production of specific nutritious food crops such as vegetables and pulses showed positive implications for an increased intake of targeted food and child nutrition (Adhiguru and Ramasamy, 2003, Hallman et al., 2003, Weinberger, 2005). Homestead gardens were found to play a crucial role in the consumption of fruits and vegetables (Adhiguru and Ramasamy, 2003). Altogether, an increased food production has been reported to be associated with a decrease in the number of underweight children (Gulati et al., 2012, Headey et al., 2012, Parasuraman and Rajaretnam, 2011) and decrease in the prevalence of low BMI (Gulati et al., 2012, Headey et al., 2012). It is not very clear whether the pathway of direct food consumption by infants and mothers or the income pathway yields this result (Headey, 2011).

The increase in food production, particularly that of staple grains, pulses and vegetables, showed more conclusive evidence on improving the nutrient intake and nutritional outcomes, compared with the overall agricultural growth rates (Adhiguru and Ramasamy, 2003, Hallman et al., 2003, Headey et al., 2012, Parasuraman and Rajaretnam, 2011, Weinberger, 2005). However, Bhagowalia et al. (2012) reported that increased food supplies facilitated the calorie intakes and improved diet diversity, but did not necessarily yield more favourable nutritional outcomes. The household and regional level estimates showed a weak relationship between calorie consumption and nutritional outcomes in India (Headey, 2011, Parasuraman and Rajaretnam, 2011).

It has been observed that households having vegetable based production systems had a lower deficiency from Recommended Daily Allowance in the consumption of vitamin A, iron, and vitamin C for adults and for children vis-à-vis those having non-vegetable based production systems (Adhiguru and Ramasamy, 2003). The interventions focused on women-operated small farm-holding households were found to yield better results, with school-age children and adolescents in beneficiary households being slightly taller than those in small farm-holding households operated by men. Nevertheless, the impact was location specific; it positively affected height for age (HAZ) scores of pre-school boys in some areas, whereas in other areas it positively affected women body mass index (BMI) (Hallman et al., 2003). Even including some of the wild and traditionally used vegetables in diet may facilitate a higher micronutrient intake and bring better nutritional outcomes (Ogle et al., 2001). Clear evidence exists that an increase in crop diversity leads to diet diversity, particularly for mothers, and improves the calorie and nutrient intake. However, evidence is not conclusive whether it results in better nutritional outcomes (Bhagowalia et al., 2012, Gaiha et al., 2012, Malapit et al., 2013).

Agricultural intensification, measured in terms of irrigation and improved seed or fertilizer use, showed a negative correlation with child nutritional outcome, particularly with respect to wasting and stunting (Shively et al., 2012).This may be caused by a shift in the cropping pattern, moving away from nutrition-rich food to not-so-nutritious cash crops, because of improved irrigation facility. This result is in concordance with the study by Parasuraman and Rajaretnam (2011), who reported that changes in cropping patterns causing crop diversity towards commercial crops may not necessarily lead to changes in the nutritional status of households. However, studies by Bhagowalia et al. (2012) and Hanji (2006) have shown that agricultural programmes aimed at improving access to irrigation significantly affect household dietary diversity and nutritional outcomes of small and marginal farmers. Therefore, no conclusive evidence exists of the effect of irrigation on nutrition.

Aquaculture appeared as a crucial source of diet diversification leading to an improved intake of animal-based micronutrients (Jahan et al., 2010, Roos et al., 2003). The farming system of integrated agriculture-aquaculture showed a positive effect on farm productivity, micro-nutrient consumption, and nutritional status (Jahan and Pemsl, 2011).

Bio-fortification of staples has been reported to be more cost effective in reducing the burden of diseases resulting from deficiencies by combating micronutrient deficiencies, as compared with fortification and supplementation, especially in developing countries (Asare-Marfo et al., 2013, Meenakshi et al., 2007). However, its impact and extent differed across countries, crops, and directed micronutrients. In the SA countries, bio-fortification was more effective as these countries are predominantly rural and some have effective seed and public distribution systems. In this region, zinc-rich cereals had the largest effect (Asare-Marfo et al., 2013, Meenakshi et al., 2007, Stein et al., 2008).

### 3.2 Pathway 2: Agricultural income

Agriculture can be leveraged to improve the nutritional outcomes indirectly through the income and expenditure routes, as perceived in this pathway, and it has been examined by eight out of the selected 25 studies. A cross-country study by Webb and Block (2012) for 29 developing countries including the countries from the SA region has reported that the structural transformation of economy may improve agricultural incomes, thereby reducing poverty, particularly in rural areas, and enhancing nutritional status. Agricultural growth had a strong and significant impact on calorie consumption of children with a weak effect on dietary diversity (Webb and Block, 2012). Although rapid economic and agricultural growth significantly contributed towards reducing stunting rates in many developing countries, it was not a sufficient condition for addressing the problem of malnutrition, because the impact of agricultural growth was location specific. Headey (2011) reported that high agricultural growth rates in some states of India, such as Gujarat, Rajasthan and Bihar, were not accompanied by a decrease in under-nutrition. Thus, it was not clear whether agricultural growth is more pro-nutrition as compared with growth in non-agricultural sectors.

The household’s main occupation may also affect the nutritional status. Nutritional security was reported to be significantly influenced by the per capita agricultural income (Kiresur et al., 2010). The agricultural households had a marginally higher stunting and wasting, than the non-agricultural households (Bhagowalia et al., 2012). The income to nutrition relationship, disaggregated by occupation, namely agricultural and non-agricultural households, revealed that stunting in children belonging to high income quantiles was lower than those belonging to the low income quantiles. This effect was more pronounced in non-agricultural households. However, non-income factors such as child vaccination and women education had a higher impact on improving the nutritional outcomes than the income.

The increase in household income through crop production at the household level had a significant positive effect on calorie intake because of the increased food expenditure (Jahan and Pemsl, 2011, Yu, 2012). The increased food expenditure coupled with demographic characteristics and lifestyle play an important role in diet diversification (Gaiha et al., 2012). Increased household wealth also significantly affected the diet diversity of children in India. As discussed earlier, no concrete evidence exists between diet diversification and nutritional outcomes. However, Parasuraman and Rajaretnam (2011) have shown a positive association between per capita expenditures on food by households and nutritional outcomes amongst children (indicated by stunting), adolescents and ever-married women (indicated by energy deficiency). Thus, there is evidence of positive impact of food expenditure on nutritional outcomes.

Considering an increasing land fragmentation in the SA region, the diversification of agriculture towards high value crops, livestock, and aquaculture has been suggested for increasing the farm income. However, the size of landholding did not show significant impact on the nutritional status (Parasuraman and Rajaretnam, 2011).

### 3.3 Pathway 3: Agricultural policies affecting food prices

Five out of the selected studies have analysed the role of agricultural policies aimed at reducing relative prices or increasing the affordability of food on the nutritional status. Therefore, the evidence base is very small. Based on the representative sample for India, it was demonstrated that policy intervention for affecting food prices played an important role in diet diversification and nutritional outcomes (Gaiha et al., 2012). The policy of improving the affordability of staples by the public distribution system provided food and nutritional security (Adhiguru and Ramasamy, 2003, Parasuraman and Rajaretnam, 2011). However, the relative price of staples has a strong and significant association with diet diversity, but not with calorie availability. In a study conducted in Bangladesh, Yu (2012) reported no evidence of increase in the price of staples (i.e., rice), leading to a decrease in its consumption because poor households try to cope up with the price shock by compromising on the expenditure on education and healthcare. Therefore, the overall health of the household members may be adversely affected due to the price rise without much effect on food intake. The poverty alleviation programmes aimed at transferring productive assets to poor farm households lead to an increase in agricultural productivity, and consequently, improve nutrition security. The interventions facilitating an increased access to agricultural credit by the rural households improved the household’s purchasing power and contributed towards enhancing the nutritional outcomes (Kiresur et al., 2010). Thus, evidence exists on the effect of reduced relative prices of staples on nutrition. However, our evidence base is very small and the studies covered under this pathway ranked low on internal validity.

### 3.4 Pathways 4–6: Empowerment and health of women

The importance of women empowerment in agriculture and its contribution to household food and nutritional security was addressed by eight studies. The nutritional status of the mothers, measured using the BMI, had statistically significant positive effects on HAZ scores and weight for age (WAZ) scores of their children aged less than three years (Bhagowalia et al., 2012). There is no conclusive evidence to indicate that women’s employment in agriculture may make them neglect their children’s care. Notably, women employed as non-agricultural unskilled labour are more likely to neglect the care of their children by leaving them in the care of elder children or other family members. However, it appears that livelihood characteristics have a higher significant effect on maternal BMI than do occupation alone (Headey et al., 2012).

Women empowerment influenced the quality of feeding practices for infants and young children, but was weakly associated with child nutrition status (Malapit et al., 2013). Agricultural interventions intended for the empowerment of women through conferring or providing land rights and autonomy to them in agricultural production might have implications on the maternal and child nutrition status (Allendorf, 2007). A significant and positive effect of land reforms on the long-term nutritional status of women and child nutrition was observed (Allendorf, 2007, Ghosh, 2007). A multivariate analysis revealed that a mother owning land halved the probability of her child being severely underweight (Allendorf, 2007). Empowerment of women, coupled with women-friendly agricultural technologies, resulted in an increased intra-household bargaining power and a larger say in household resource allocation. This may eventually lead to the provision of nutritious food for themselves and their children (Hallman et al., 2003, Allendorf, 2007, Ghosh, 2007). An effective participation of women in the nutritional programme requires a gender sensitive approach, particularly in aquaculture (Jahan and Pemsl, 2011). Improving nutrition knowledge, particularly of women, had a strong association with food production, consumption, and expenditure on food (Adhiguru and Ramasamy, 2003, Gulati et al., 2012) and thus seems to be crucial for nutritional outcomes as it runs through all the pathways.

An index of ‘Women’s Empowerment in Agriculture’ (WEAI), combining five domains of empowerment sub-index and a gender-parity index, showed a significant and positive association with calorie availability and household dietary diversity (Malapit et al., 2013, Sraboni et al., 2013). It had different effects on dietary diversity and nutritional outcomes for women and children depending on the dimension of empowerment. Women’s autonomy in agricultural production decisions was one of the major factors having positive effects on maternal and child nutritional outcomes, except for maternal BMIs. However, women’s control over the household income improved child WAZ scores and maternal BMIs. A significant positive effect was observed on the number of hours women were engaged in paid and unpaid agricultural work, children’s diet diversity, and children’s WHZ and HAZ scores. The association with Women’s Group and narrowing of gender gap had a significant positive effect on household food security (Sraboni et al., 2013, Malapit et al., 2013) and maternal BMIs (Malapit et al., 2013). All these studies establish the validity of the women empowerment pathways.

## 4 Discussion

The set of studies selected for review, although heterogeneous in terms of agricultural interventions and nutritional outcomes, shows an association between various interventions and outcome indicators through empirical techniques. The review confirms that agricultural interventions targeted at nutrient-rich crops and diversification of agricultural production system towards fruits, vegetables, and aquaculture through women empowerment hold the potential to improve the nutritional outcomes in South Asia.

The main feature of our review exercise is a systematic approach which involved assessing the internal and external validities of the studies under review. The quality of evidence constrained the exercise to 25 studies. Only one of these studies ranked high on the internal validity. None of the included studies used RCT for studying the effect of interventions. Hence, the existing indications cannot be interpreted as a concrete evidence of the nutritional impact of agricultural interventions (Kadiyala et al., 2014). The gaps in the model design and empirical testing could be the possible reasons for the statistical techniques not being able to establish substantial associations (Masset et al., 2011, Webb and Kennedy, 2014).

The studies on agriculture for improved nutrition in South Asia have generally focused on the first and second pathways. Very few of them have covered a relative price policy (pathway 3) and women empowerment (pathways 4–6). The studies typically included evaluations of specific agricultural interventions, analysis of different data sets for change in consumption pattern, and calorie and nutrient intake to capture the nutritional outcomes. The measurement of anthropometric factors and the micronutrient level as direct outcome indicators has been scanty, as also mentioned by Haen et al., 2011, Hawkes et al., 2012 and Kadiyala et al. (2014).

**Fig. 3:**
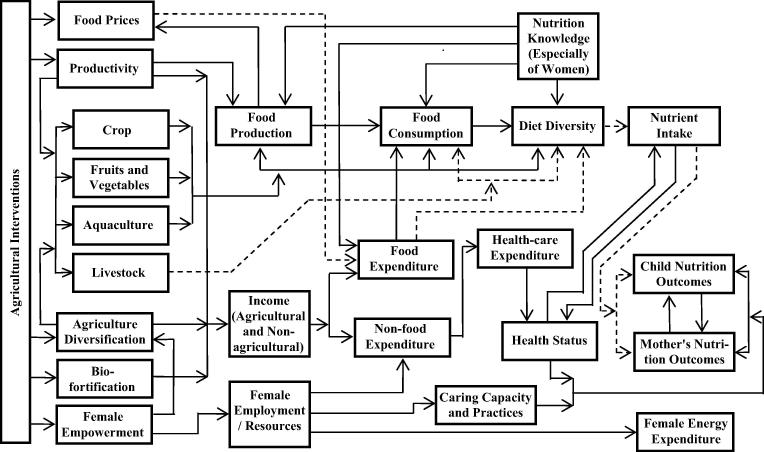
Pathways of agricultural interventions and nutrition.

The major pathways linking agriculture with nutrition and illustrating the evidences are shown in Fig. 3. The pathways supported by strong evidence are marked as solid lines and those with weak evidence by dotted lines. Strong evidence exists for the first pathway. It indicates that agricultural interventions for increasing the productivity and crop diversification are successful in promoting targeted food production and consumption, leading to dietary diversity and the intake of specific nutrient types. The role of homestead garden seems to be very important in improving diet diversification, although the evidence base for enhanced nutritional outcomes is weak. No conclusive evidence exists for the impact of irrigation on nutritional outcomes. In the South Asian context, these agricultural interventions need to be supplemented with bio-fortification and nutrition knowledge for addressing the socio-economic needs of landholders, enabling them to act as vehicles for nutritional enhancements of children and women. These findings show the importance of home production of nutrition-rich food crops for improved diet and the results differ from those reported in the reviews by Kadiyala et al. (2014) for India and Yosef et al. (2015) for Bangladesh. Integrated agriculture-aquaculture appears to be important for dietary diversity and nutritional outcomes. The results on the role of agricultural diversification and homestead garden, but not those on the roles of irrigation and livestock ownership, are in accordance with the previous reviews.

The review further shows that agricultural growth is not a sufficient condition for addressing the problem of malnutrition, because its impact is location specific and depends on the state of economy and other socio-demographic conditions. Income and under-nutrition are weakly associated, whereas non-income factors, such as child vaccination and women education, have strong and significant effects. The pathway of agricultural policies affecting the affordability has positive effect on food security at the household level; however, its effect on diet diversity and nutritional outcomes is not conclusive.

The pathways of women in agriculture and their empowerment (pathways 4–6) have poor evidence base, but they bring out the importance of women employment, land rights and access to resources, for improving the nutritional status of the mothers and their children. The evidences are consistent across the studies included in the review. They show the benefit of reducing the gender gap and the empowerment of women for improving the nutritional status, as reported earlier (Berti et al., 2004, Kadiyala et al., 2014). Our findings regarding the role of nutrition knowledge is in accordance with previous reviews (Ruel, 2001, Haider and Bhutta, 2008, Girard et al., 2012). Thus, the agricultural interventions with a multi-sectoral approach and targeted at empowering women can enhance the nutritional outcomes for women and children.

## 5 Conclusions

The agricultural interventions have the potential to influence nutritional outcomes in the South Asia; however, the available evidence linking the agricultural interventions and their impact on the nutritional status of women and children is small. The findings, generally in concordance with those from the previous reviews, show that linkages between agriculture and nutrition are complex and require multi-sectoral and multi-dimensional approaches to tackle the malnutrition problem in this region. However, the findings clearly indicate the importance of the home production of nutrient-rich food crops for improving the nutritional outcomes. Bio-fortification of staples and homestead gardens can influence the intake of a micronutrient-rich diet and consequently nutritional outcomes. The diversification of agriculture towards fruits and vegetables and integrated agriculture-aquaculture can potentially promote dietary diversity and improve nutritional outcomes. With more favourable nutrition-sensitive agricultural policies and empowerment of women, it is possible to improve nutritional status.

There exists a need for well-designed studies with sound methodologies and rigorous analytical techniques, as indicated in some earlier reviews (Kadiyala et al., 2014, Masset et al., 2011, Webb and Kennedy, 2014). Such studies can facilitate substantial inferences on the linkages between agriculture, intermediate outcomes, and nutritional status. The data gaps for linking agriculture and nutrition have been previously identified (Haen et al., 2011, Kadiyala et al., 2014), and integrated datasets are required for understanding these linkages to leverage them for improving nutritional outcomes.

## Figures and Tables

**Table 1:** Results of advance search with Google Scholar for the period 2000–2014.

With all words	Exact phrase	No. of papers
Agriculture nutrition	–	1170625
Agriculture nutrition South Asia	–	72514
Agriculture nutrition South Asia	Agriculture Nutrition	2080
Agriculture intervention nutrition South Asia	Agriculture Nutrition	1150
Agriculture programme nutrition South Asia	Agriculture Nutrition	1620
Agriculture programme nutrition impact South Asia	Agriculture Nutrition	1600
Impact Agriculture intervention nutrition South Asia	Agriculture Nutrition	1140
Effect Agriculture intervention nutrition South Asia	Agriculture Nutrition	1150
Impact Agriculture intervention nutrition outcome South Asia	Agriculture Nutrition	1100
Effect Agriculture intervention nutritional outcome South Asia	Agriculture Nutrition	865
Impact Agriculture intervention nutritional outcome South Asia	Agriculture Nutrition	863

**Table 2:** Scoring criteria for internal and external validity.

Criteria used	Low score	Medium score	High score
*(a) Internal Validity*			
Counterfactual analysis	• Weak or no comparisons of participants to unmatched non-participants • No control group	• Control group may have been used • No difference-in-difference analysis	• Control group vs. non-control group carried out • Difference-in-difference analysis conducted

*(b) External Validity*			
Programme theory	• No programme theory presented • No intermediate outcomes analysed	• Programme theory presented • Intermediate outcomes considered but not analysed Or • No intermediate outcomes considered	• Programme theory presented • Intermediate outcomes estimated and analysed
Heterogeneity of impact	No heterogeneity of impact undertaken	Heterogeneity of impact mentioned but no analysis undertaken	Heterogeneity of impact analysis undertaken

**Table 3:** Empirical studies included in the study.

Sr.	Study	Outcome measure	Area of research	Internal validity	External validity		Main findings
Counterfactual score	Programme theory	Heterogeneity of impact
1	Adhiguru and Ramasamy (2003)	RDA for women and children using 24 h recall method	India, Primary survey data – 180 households from Dharmapuri district, TamilNadu	Low	Medium	Medium	• Vegetable cultivation has immense potential to supply vitamin rich foods and micronutrients to weaker sections • Vegetable consumption is higher in the households producing vegetables • Nutrition knowledge, especially in women, is very important • PDS serves the purpose of nutrition security • Homestead gardens increase consumption of fruits and vegetables

2	Allendorf (2007)	Anthropometric– children (severely underweight)	Nepal, Demographic and Health Survey 2001, 8,633 households, agricultural workers	Low	High	High	• Women’s empowerment is important for the agriculture nutrition link • Women’s land rights empower women, benefit family welfare and child health and nutritional status (underweight)

3	Asare-Marfo et al. (2013)	Biofortification	7 staple crops across 127 countries in Asia, Africa, Latin America and the Caribbean (LAC)	Low	Medium	Low	• For Asian countries, zinc-rich cereals (rice and wheat) have the largest effect • Iron bio-fortification of pearl millet in some South Asian countries

4	Bhagowalia et al. (2012)	Anthropometric outcomes (HAZ and WHZ for children 0–5 and 8–11 years)	India, India Human Development Survey 2005	Low	Medium	High	• Agricultural income and production conditions have significant influence on household dietary diversity • Agricultural programs aimed at irrigation, livestock ownership, and crop diversification have significant impact on dietary diversity • Stunting and wasting rates are marginally higher for agricultural households vis-à-vis non-agricultural households • Children belonging to the highest income quantiles have higher HAZ vis-à-vis the poorest quantile, this effect is stronger for non-agricultural households • Income gradient for under-nutrition is weak while non-income factors such as child vaccinations and female secondary education have strong significant effects on reducing malnutrition • Crop diversity is positively associated with diet diversity

5	Gaiha et al. (2012)	Diet diversification	India, 1993–2009, NSS 50th, 61st and 66th Rounds unit record data over 3 NSS years	Low	Medium	High	• Dietary shifts are associated with more than moderate reductions in calorie intakes, i.e. taste for food variety, leading to lowered calorie intakes • Food prices, expenditure, demographic characteristics and lifestyle play important roles in diet diversification and nutritional outcomes • Small and marginal farmers have higher contribution in calorie, protein, and fat production; but profit earned is low due to poor marketing infrastructure

6	Ghosh (2007)	Anthropometric – women’s height and HAZ for children	India, NFHS – II 1998–99 sample size of 67,600 women, 16 major states	Medium	High	Low	• Land reforms, especially reforms targeting abolition of intermediaries and imposition of land ceilings, lead to significant improvements in women’s long term nutritional status (or height) and also have a bearing on child nutritional attainments

7	Gulati et al. (2012)	Normalized malnutrition index using anthropometric outcomes (children <5 years and adults) WAZ, HAZ, WHZ, and BMI	India, NFHS – III, 2005–06	Low	Medium	High	• Improvements in agricultural productivity can be a powerful tool to reduce under-nutrition in adults and children • Malnutrition is a multidimensional problem:Aaccess to sanitation facilities and women’s literacy are strong factors affecting malnutrition

8	Hallman et al. (2003)	Anthropometric- height for age- HAZ, adult female BMIs and calorie intakes	Bangladesh, 955 Rural households	Low	Medium	High	• Vegetable technology targeted at women in households of small landholdings has positive effects on female empowerment and child nutritional status • Group fishpond technology is highly beneficial for poor households leading to higher off-farm incomes and improved nutritional status • Regressions for nutritional status for adults and children show no effects of fishpond technologies in the pooled sample; but the access towards technological advancements has strong significant effects on pre-schooler HAZ • Non-lumpy technology has more positive effect on nutritional outcomes of children and women empowerment

9	Hanji (2006)	Anthropometric – adolescent girls and adult BMIs	India, 192 households from 8 villages in 3 talukas of Belgaum district, Karnataka,	Medium	High	Low	• Irrigation facilities leads to a shift in cropping patterns in favour of high value crops and improves nutritional intake

10	Headey (2011)	Anthropometric outcomes (children - stunting) WAZ, HAZ and BMIand diet diversification	Cross-country dataset	Low	Medium	High	• Agricultural growth has larger effects in reducing malnutrition as compared to non-agricultural growth • Agricultural growth has a strong effect on daily energy supply (calorie consumption) but a weak effect on nutritional outcomes • Relationship between agricultural growth and malnutrition is heterogeneous • Agricultural growth has insignificant effect on malnutrition in Indian states but it has a highly negative effect in other developing countries (specifically for stunting)

11	Headey et al. (2012)	Anthropometric (BMI for women and HAZ and WAZ)	India, DHS 2009, Indiastat, RBI (2010, FAO (2009), NFHS – II	Low	High	High	• There is no positive association between calorie consumption and nutritional outcomes at both household and regional level • Mother’s BMI has effect on child stunting but there is no conclusive evidence for underweight • Ceteris paribus, BMIs for female agricultural workers are lower than for female non-agricultural workers with livelihood characteristics having an important negative bearing on adult BMIs • Female employment in agriculture has significant but small positive effect on HAZ scores for children • Evidence doesn’t corroborate the hypothesis that child care practices are poorer in agricultural households or by agriculturally employed mothers

12	Jahan and Pemsl (2011)	Consumption pattern change	Bangladesh, impact of long-term training provided to small-scale farmers	Medium	High	High	• Integrated agriculture- aquaculture (IAA) has a significant positive impact on fish consumption • Significant positive effect on fish consumption could perhaps result from either higher level of fish farming inputs purchased using grant money, or a general higher overall interest in fish farming or project activities • IAA increases agricultural diversity, agricultural productivity, and food consumption

13	Jahan et al. (2010)	Consumption pattern change	Bangladesh, 2002–03 and 2003–04, 225 farmers	Low	High	Medium	• Aquaculture interventions have positive effect on consumption and household nutrition • Aquaculture interventions can bring about reduction in poverty and improvements in nutritional status of resource poor households • Women’s effective participation and access to nutritional benefits can be facilitated through gender sensitive approach in aquaculture

14	Kiresur et al. (2010)	Consumption pattern change	India, Bagalakot district, Karnataka, 120 farm households, 2005–06	Medium	Low	Low	• Nutritional security of respondents is significantly influenced by agricultural income per consumer unit per annum, literates/ household, total consumer units per household • Enhancements in agricultural productivity through transfer of productive assets to poor households and increased access to agricultural credit by rural households would contribute towards enhancing nutritional status

15	Malapit et al. (2013)	Anthropometric – mother’s BMI and children <5 years HAZ, WAZ, HWZ,	Nepal,Household survey data conducted in 4,080 households across 16 districts	Low	Medium	High	• Production diversity at household level determines maternal nutrition outcomes, mother’s dietary diversity and BMI. For children, this effect seems to be facilitated through the age of the child • Autonomy in agricultural production decisions as a measure of women empowerment is a key determinant of almost all mother and child outcomes with the exception of maternal BMI • Women’s empowerment influences quality of infant and young child feeding practices and weakly associated with child nutrition status

16	Meenakshi et al. (2007)	Disability adjusted life years (DALYs)	12 countries in Asia, Africa and Latin America	Low	Medium	Low	• Bio-fortification can have positive effects in terms of reducing micronutrient deficiencies and it is a cost-effective intervention • Bio-fortification is more cost-effective than supplementation or fortification • In South Asian countries, bio-fortification is more effective since these countries are predominantly rural and have effective seed distribution systems in place

17	Ogle et al. (2001)	Diet diversification	Vietnam, two villages, Rural 217 women	High	Low	Low	• Wild vegetables make a significant contribution to overall micronutrient intakes viz., carotene, vitamin C and calcium • Analysis of food variety helps in bringing forth the benefits of wild vegetables

18	Parasuraman and Rajaretnam (2011)	Anthropometric for children HAZ, WAZ (<5 years) and BMI for adolescent girls and women	India, Vidarbha region, Maharashtra, primary survey, 6990 households in six high distress districts	Low	Medium	High	• Cultivation of food crops contribute towards improvements in child nutrition • Ceteris paribus, higher the expenditure on food items, lower the proportion of undernourished children, adolescents and ever married women • Visible changes in agricultural cropping patterns cannot be taken as indicators of better nutritional status of households • PDS contributed significantly to increase food security • Consumption of food from own production reduces child stunting and underweight

19	Roos et al. (2003)	Diet diversification	Bangladesh, 84 poor rural households in Kishoreganj district (June 1997- January 1998)	Low	Medium	Low	• No difference in fish intake in the fish producing and non-fish producing control households • Fish consumption contributed to <10% of required protein intakes • Production of vitamin A: dense SIS (small indigenous fish species) can make important nutritional contributions

20	Shively et al. (2012)	Anthropometric outcomes (children <5 years). HAZ and HWZ by agro ecological zones	Nepal, Demographic and Health Survey 2001, 10,793 women, 4397 men (age 15–59) and 5,464 children <5 yrs	Low	Medium	High	• Agriculture is important for child nutrition • Agricultural intensification, including fertilizer, irrigation, and use of improved seeds, leads to lower HAZ and WHZ scores

21	Sraboni et al. (2013)	Per adult equivalent calorie availability and dietary diversity	Bangladesh, BIHS data, 3944 households	Low	Medium	High	• There is a significant association between crop diversity and diet diversity • There is no significant relationship between crop diversity and calorie availability • There is positive associations between household calorie availability and dietary intakes and women’s empowerment score, number of groups in which women participate, women’s control over assets • Empowerment of women in leadership in community and control over resources was lower • Relative price of staple food has strong and significant association with diet diversity but not with energy availability • Ownership of cultivated land was associated with household energy availability and diet diversity

22	Stein et al. (2008)	Disability Adjusted life years (DALYs)	India, Iron bio-fortification	Low	Medium	Low	• Under pessimistic assumptions, iron bio-fortification of rice and wheat could save 0.8 million DALYs annually • Under optimistic assumptions, the DALYs saved could be 2.3 million • Sizeable health benefits can be reaped when iron content of rice and wheat is enhanced and its coverage increased • Iron bio-fortification of rice and wheat is a very cost-effective agricultural intervention

23	Webb and Block (2012)	Anthropometric – children less than 5 years of age, HAZ, WAZ, and WHZ	Developing Countries, Panel for 29 developing countries, 1980–2007	Low	Medium	Low	• Backing agriculture and poverty reduction strongly support reduction in child under-nutrition (stunting and wasting) • Agriculture support increases rural incomes faster and decline in under-nutrition is more pronounced in rural settings

24	Weinberger (2005)	Consumption pattern change	Pakistan, primary survey around Lahore area, industries employing females on a piece rate basis, June 2001-February 2002	Medium	Medium	Low	• Agriculture plays important role in reducing malnutrition by increasing agricultural productivity • Bio-availability of iron from mung bean is high • Increase in pulses (mung bean) productivity has substantial effect on nutrition, iron intake, and human productivity • Policy to increase availability of targeted crop is needed for improving the nutrition outcome

25	Yu (2012)	Consumption pattern change	Bangladesh, IFPRI Chronic Poverty and Longer Term Impact Study, 1237 households, 50 villages in 2005/06	Low	Medium	High	• Nutrition is governed by household size, characteristics of household head, asset ownership, consumption of own produce • There was no evidence that increase of rice price reduces rice intake – as household cope up by reducing expenditure in education and health care • Female headed households face difficulties in meeting their nutrition needs • Increase in rice yield through agricultural research and development is an effective way of improving nutrition • Specific policies are needed to address the food and nutrition needs of vulnerable
